# Dealing with Noisy Absences to Optimize Species Distribution Models: An Iterative Ensemble Modelling Approach

**DOI:** 10.1371/journal.pone.0049508

**Published:** 2012-11-15

**Authors:** Christine Lauzeral, Gaël Grenouillet, Sébastien Brosse

**Affiliations:** 1 Laboratoire Évolution et Diversité Biologique, UMR 5174, Université de Toulouse, UPS, ENFA, Toulouse, France; 2 UMR 5174 EDB, CNRS, Toulouse, France; University of Western Australia, Australia

## Abstract

Species distribution models (SDMs) are widespread in ecology and conservation biology, but their accuracy can be lowered by non-environmental (noisy) absences that are common in species occurrence data. Here we propose an iterative ensemble modelling (IEM) method to deal with noisy absences and hence improve the predictive reliability of ensemble modelling of species distributions. In the IEM approach, outputs of a classical ensemble model (EM) were used to update the raw occurrence data. The revised data was then used as input for a new EM run. This process was iterated until the predictions stabilized. The outputs of the iterative method were compared to those of the classical EM using virtual species. The IEM process tended to converge rapidly. It increased the consensus between predictions provided by the different methods as well as between those provided by different learning data sets. Comparing IEM and EM showed that for high levels of non-environmental absences, iterations significantly increased prediction reliability measured by the Kappa and TSS indices, as well as the percentage of well-predicted sites. Compared to EM, IEM also reduced biases in estimates of species prevalence. Compared to the classical EM method, IEM improves the reliability of species predictions. It particularly deals with noisy absences that are replaced in the data matrices by simulated presences during the iterative modelling process. IEM thus constitutes a promising way to increase the accuracy of EM predictions of difficult-to-detect species, as well as of species that are not in equilibrium with their environment.

## Introduction

The ability to predict species distributions is a prerequisite to anticipate environmental changes and to set up sound conservation priorities. There are basically two types of species distribution models (SDMs): mechanistic (or process-based) models that are based on physiological and ecological characteristics of the species and correlative (or niche-based) models that build predictions on the basis of observed species-environment relationships [1]. Mechanistic models require a detailed knowledge of the species considered and are therefore used to predict the distribution of well-known species (e.g., of high conservation or economic value) [reviewed in 2]. In contrast, correlative SDMs are based on the generalization of observed species-environment relationships, and can hence be applied to a large number of species [e.g., 3,4].

Presence-absence data are the most commonly used to feed SDMs as such data are often available over larger areas than species abundances. Although the presence of a species is factual, absence can have a multiple meaning. Lobo *et al.* [5] listed three distinct types of absences: environmental absences (the environmental conditions do not allow the presence of the species), contingent absences (the environmental conditions are favorable but other factors such as biotic interactions, barriers to dispersion or local extinction are responsible for the absence of the species) and methodological absences (the species is present but not detected). Unlike environmental absences, contingent and methodological absences are noisy absences known to reduce the reliability of SDMs predictions of the potential niche of the considered species, i.e. the range of environmental conditions where the species can be present [6]. Indeed, although contingent absences are informative to define the realized niche (i.e. the environmental conditions where the species is really present) they drive SDMs to an underprediction of the potential niche of the species. Moreover methodological absences are always uninformative and blur both potential and realized niche predictions. In the context of applied ecology, prevention plans against invasion as well as threatened species conservation plans often require the identification of the potential niche of the species to predict how their niche could become extended (for invasives) or reduced (for threatened species) under various scenarios. In such a potential niche prediction context, both methodological and contingent absences are considered as noisy absences.

To account for potential sampling errors and distinguish between non-detection and true absences, binomial likelihood models have been used to estimate changes in range boundaries under recent climate change [7], [8] and to correct site-occupancy models for imperfect detection [9]. Although these models are efficient, they are designed to be computed using species abundance data or the detection/non-detection pattern at sites surveyed at least twice [9]. In the same way, Galien *et* *al.* [10] proposed to combine global- and regional-scale data to deal with non-environmental absences and improve the accuracy of SDM prediction of the potential distribution of invasive species. However, this design is only applicable when both large and small scale data are available, which is not the case for most species.

Presence-only SDMs offer another alternative to the problem of absence uncertainty, as they only consider the presence of the species to determine its niche [11], [12]. Their performance however remains lower than presence-absence SDMs [13] and they frequently overestimate potential distributions compared with presence–absence models [14].

In order to use presence-absence models when no reliable absence data are available, the use of “pseudo-absences”, i.e. of environmental conditions available in the studied area and assumed to be absence points, has also been suggested [14]. “Pseudo-absences” can be simulated through various strategies (e.g. pseudo-absence selection from low suitability areas predicted by a presence only model [15]; pseudo-absence weighted using the outputs of a model built at a larger scale [10], pseudo-absence selection as sites distant from presence sites, but it remains unclear how those strategies affect the models [14]–[18], so the use of randomly generated pseudo-absences is often encouraged [13], [18], [19]. Such random selection of absences can however reduce model accuracy, leading to an overestimation of the actual range of the species through the selection of uninformative absences, as well as an underestimation of the range through the selection of non-environmental absences (Lobo 2010). In addition, both presence-only models and presence-absence models built using pseudo-absence neglect the ecological information contained in the environmental absence data, making presence-absence models built using true absences more accurate [13].

A wide range of correlative models using presence-absence data have been developed since the nineteen eighties. They are based on various statistical techniques ranging from regression (e.g. multiple linear regression, generalized additive models) to classification (e.g. classification and regression trees, linear discriminant analysis) and machine learning (e.g. artificial neural networks, boosted trees). These techniques have been shown to vary considerably in both performance and spatial predictions of species distributions, and despite an abundant literature on method comparisons, no consensus has emerged as to the most suitable statistical method [20]–[23]. In view of this variability between predictions of SDMs, the recommendation is thus to simultaneously apply a wide range of statistical methods, all built using the same environmental data, and to produce a consensual response that synthesizes individual model outputs, giving rise to ensemble modelling ([ensemble modelling, EM, 24,25]). EMs have increasingly been used these last years as they are recognized to provide significantly more robust predictions than all the single models [26] and to perform better than single SDMs as EMs buffer individual bias of each method and hence enhance prediction reliability [25], [27]. Disentangling environmental and non-environmental absences in EMs might thus constitute a promising way to enhance the reliability of the prediction of species potential distribution.

Here we propose an optimization of EM by using an iterative ensemble model (hereafter called IEM), designed to reduce the effect of noisy absences in potential niche prediction. To do this, we considered noisy absences to be the false presences predicted by the model (i.e., commission errors, when the model predicted species presence while it was actually absent from the training set). These noisy absences were then considered as presence and the resulting new data matrix was used as a new model training set. This post-processing of model outputs was iterated until the predictions stabilized, therefore providing a potential distribution of the species. Such a strategy presents some similarities with the pseudo-absences selection methods [15], but differs by two main points: firstly, it is only based on the use of presence-absence models that are known to be more efficient than presence-only models [14]; secondly, the noisy absences are not discarded but converted into presences.

**Figure 1:**
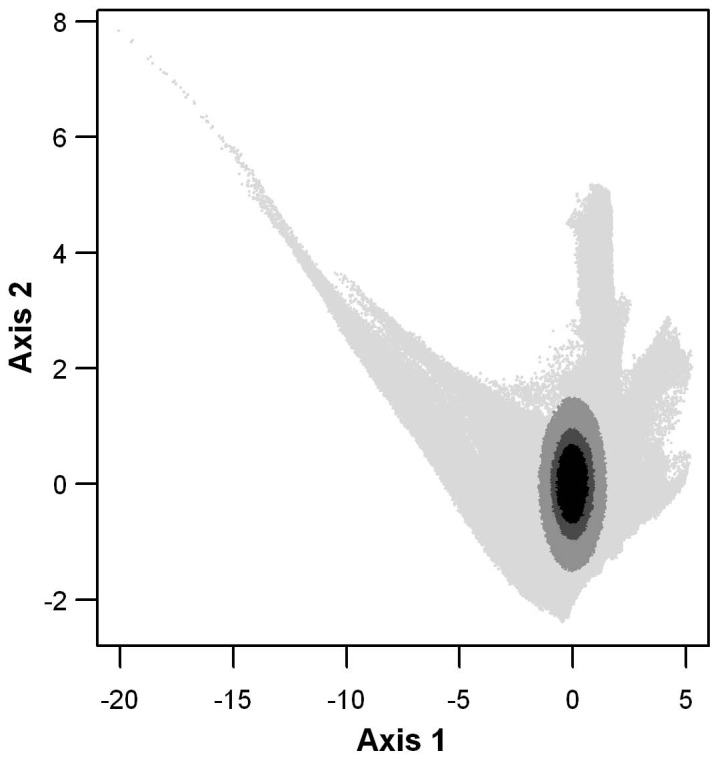
Figure **1. The niches of the 3 virtual species in the two-dimensional space created by the two orthogonal axes summarizing climatic variation across France.** Prevalence 15% (black), 30% (dark grey) and 60% (grey).

In this context, the main objectives of this study were: (i) to compare the performances of EM and IEM to predict the spatial distribution of individual species and (ii) to assess the ability of the two modelling methods to deal with noisy absences. To do this, we used eight climatic variables to construct the potential climatic niche of three virtual species over France. For each species, we introduced non-environmental absences into the simulated occurrence data in two ways: a random distribution and a distance gradient from the center of the environmental niche. In this last case, the occurrence of non-environmental absences was maximal at the edge of the environmental niche, where the species density usually decreases [28] making the species less detectable.

## Materials and Methods

### Predictor Variables

Eight climate variables were extracted over France from the 30′′×30′′ resolution WorldClim layers for the period 1961–1990 [29]: precipitation in the driest quarter of the year and in the wettest quarter; average monthly precipitation and precipitation seasonality; mean temperature of the coldest quarter and of the warmest quarter, annual mean temperature and temperature seasonality. These variables were chosen as they are related to the ecological requirements of numerous species, and have often been used in SDMs [30]–[32].

### Virtual Ecological Niches

The virtual species distributions were defined as hyper volumes of a space defined by a set of relevant environmental variables [33], [34]. A normalized principal component analysis (PCA) was computed on the eight climate variables and the first two axes of the PCA, which accounted for 80% of the total variance, were kept as composite variables that summarize the climate data. We hence constructed two independent climate variables [34]. In the two-dimensional space created by the two orthogonal axes summarizing climatic variation across France, the virtual species niches were defined as discs [35] centred on (0,0) (Fig. 1). All geographic cells falling within this disc for the pair of climate variables were considered as the observed distribution range of the virtual species in France. Using three different disc radii, three virtual species were created, with prevalences of 15%, 30% and 60% respectively so as to cover a large prevalence range.

**Figure 2:**
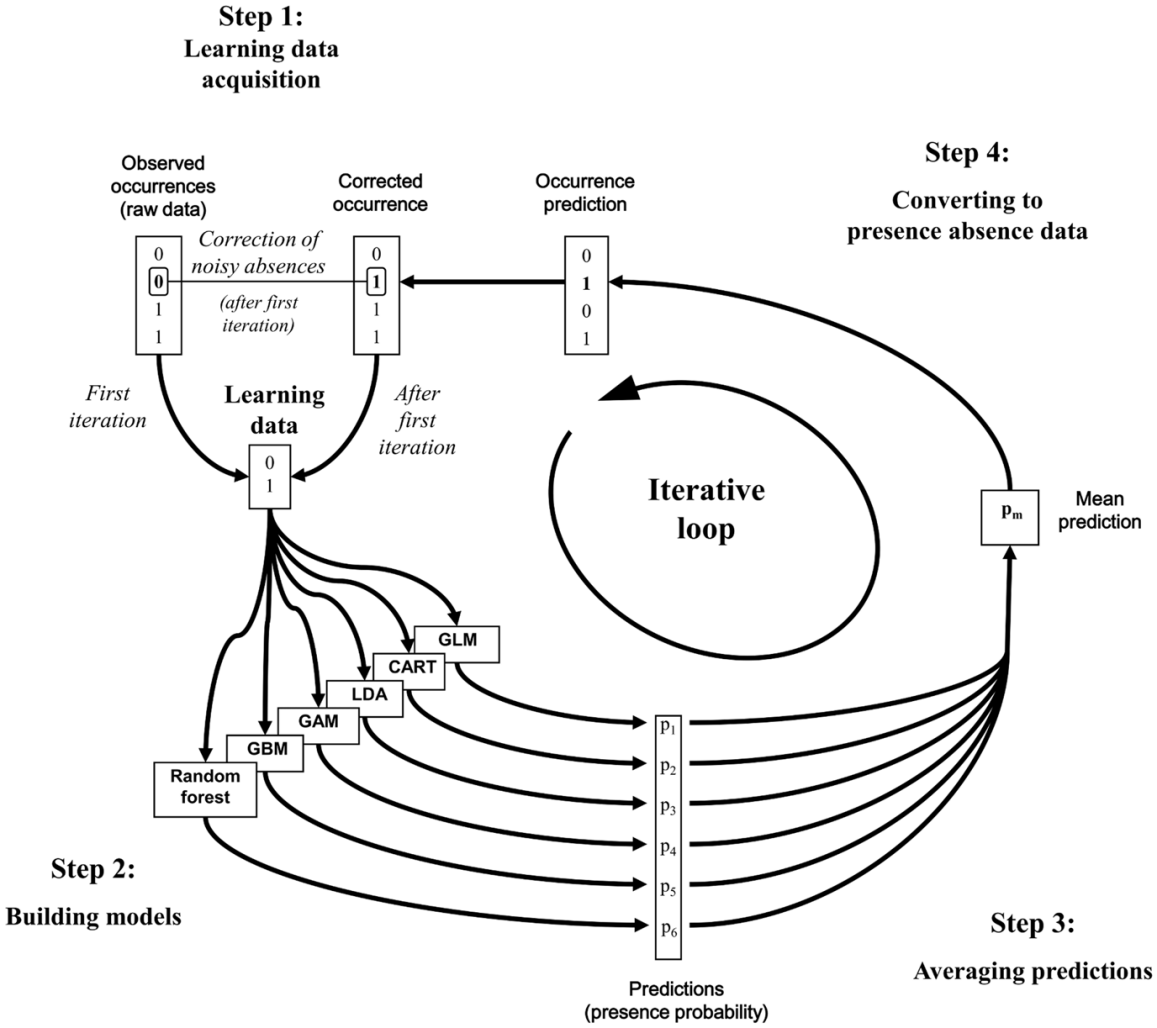
Figure **2. The iterative ensemble modelling (IEM) process.** Step 1: At the first iteration, the learning data is the original data set with n = 666 sites. For the following iterations, the learning data is the raw data set updated using the predicted data matrix: an absence is considered as noisy if the model predicts presence while the species is absent from the observed data. In that case, the raw data is updated by replacing absence (0) by presence (1); Step 2: The six statistical methods are used to build models with the learning data set; Step 3: the six resulting suitability levels for each site (one per modelling method) are averaged, giving rise to a per-site suitability level; Step 4: the suitability vector is converted into a presence-absence response, using a cut-off threshold maximizing the Kappa index.

### Data Sets

First, 1000 cells were randomly sampled among the 912730 cells covering the entire surface of France. These 1000 cells were considered as the sampling sites. This operation was repeated 100 times, giving rise to 100 data sets. Each of these 100 data sets was randomly split into two parts: two-thirds of the data (666 sites) were used to calibrate the SDMs and the remaining third (334 sites) was used as a test set.

**Figure 3:**
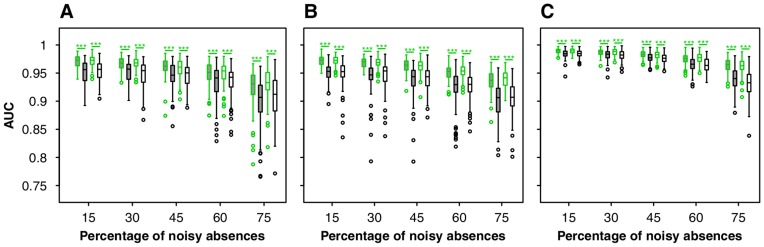
Figure **3. Effects of noisy absences on threshold-independent measurements (i.e., AUC) of model accuracy after the first iteration (EM, in green) and at the end of the process (IEM, in black) for three virtual species with true prevalence of (A) 15%, (B) 30% and (C) 60%.** Box colors represent geographic distribution of noisy absences (grey: random; white: mostly at the edge of the niche).

Then, five occurrence levels of noisy absences (15%; 30%; 45%; 60% and 75% of all the presences available in the learning data set) were inserted into the learning data set. For each occurrence level, two strategies were used to determine the position of the noisy absences. On the one hand, noisy absences were selected randomly from all the presences available in the learning data set. On the other hand, we assumed that the probability of a site inside the niche to be a noisy absence increased as a Gaussian function of the distance to the centre of the environmental niche. More explicitly, the probability of the site being selected was equal to (1–0.9 exp^(-d^2^/r^2)^^)/n where d was the distance to the centre of the environmental niche, r was the radius of the environmental niche and n was chosen to ensure that the sum of the probabilities over all presence sites is equal to 1. We thus obtained 1000 (5 noisy absence percentages, 2 absence distribution types, 100 repetitions) data sets for each species.

**Figure 4:**
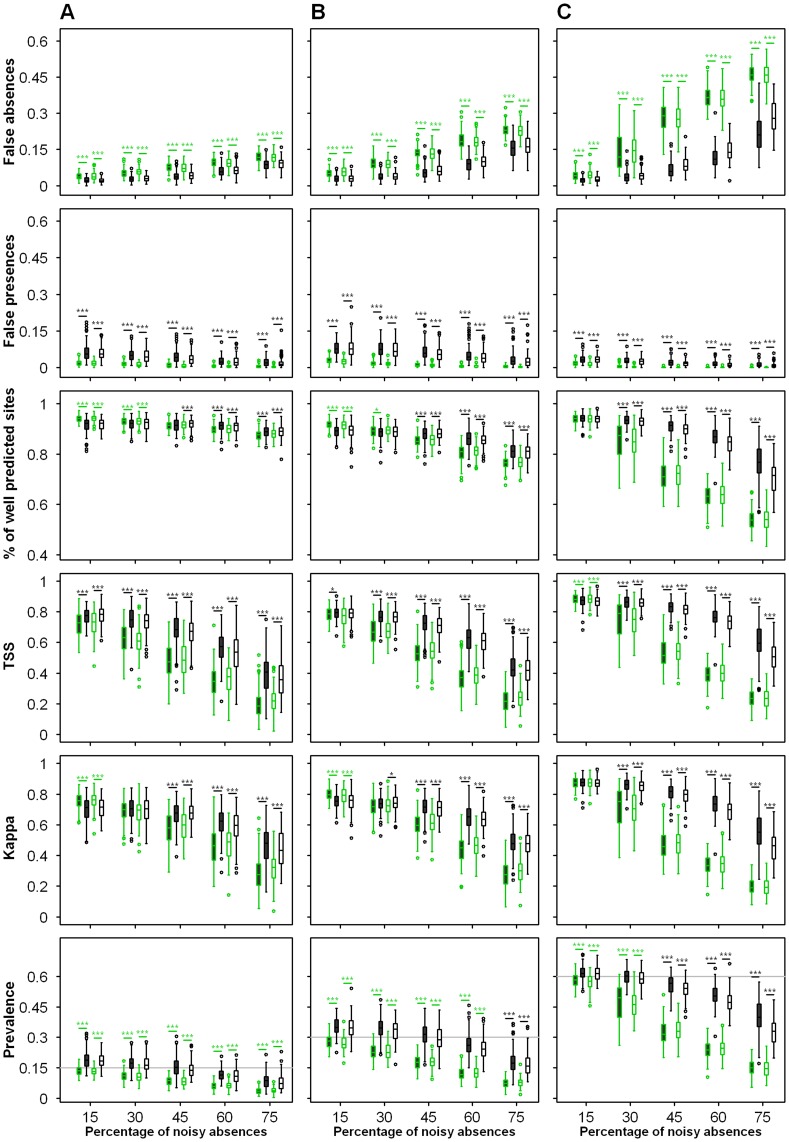
Figure **4. Effects of noisy absences on threshold-dependent measures of model accuracy after the first iteration (EM, in green) and at the end of the process (IEM, in black) for three virtual species with true prevalence of (A) 15%, (B) 30% and (C) 60%.** Model accuracy was evaluated using the two types of mispredicted sites, percentage of well-predicted sites, TSS, Kappa, and predicted prevalence. Box colors represent geographic distribution of noisy absences (grey: random; white: mostly at the edge of the niche). The grey line corresponds to the true value of the prevalence.

### IEM Modelling

According to the EM framework, we used six predictive modelling methods belonging to 3 commonly used correlative SDM techniques, and hence balance the weight of each technique and its inherent biases. These methods included two regression techniques: generalized linear models (GLM); generalized additive models (GAM); two machine learning techniques: Random Forest (RF) and generalized boosted regression models (GBM); and two classification techniques: classification and regression trees (CART); linear discriminant analysis (LDA). Raw variables were used without prior transformation in all models, squared variables were included in GLM and LDA models to deal with non-linearity. For the GAM model all the variables were spline-transformed (df = 4). In GBM, a maximal number of 1000 trees was generated. In RF, 300 trees were generated and the number of predictors randomly selected at each node was the square root of the total number of predictors. Those six methods have already been used in different EM studies [26], [27], although there is no strict consensus on which method should be implemented in each EM.

**Figure 5:**
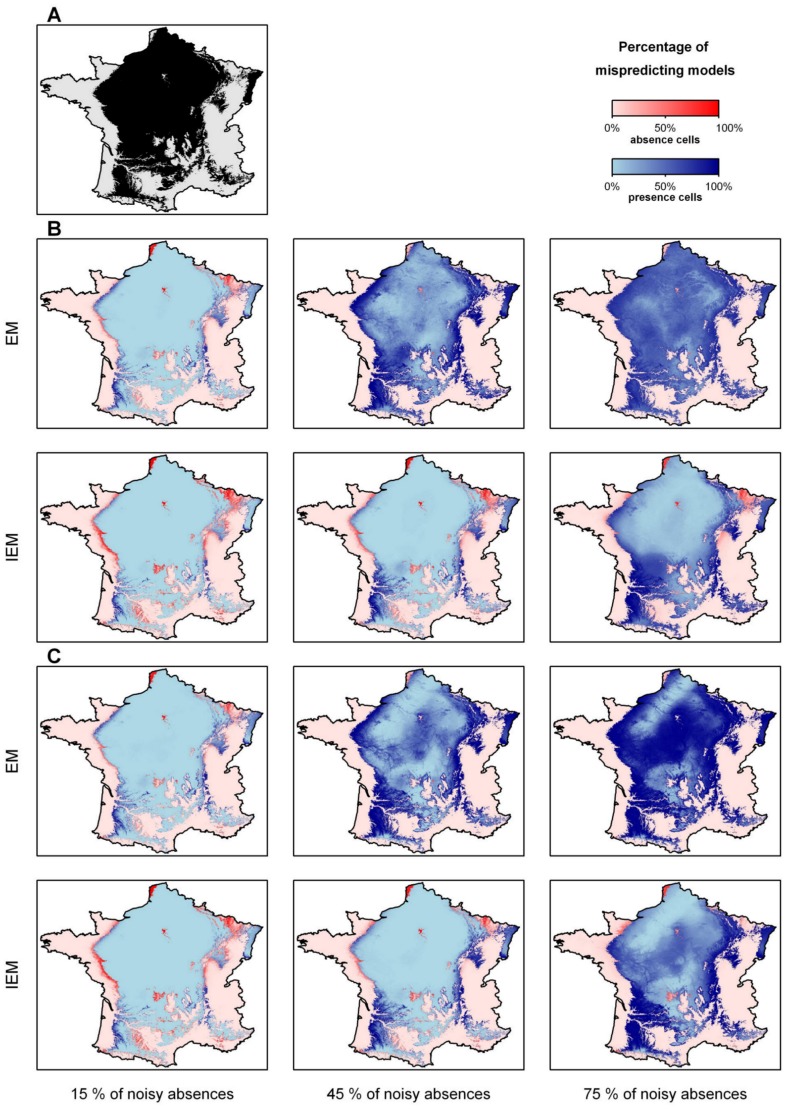
Distributions of the frequent species (prevalence = 60%). (A) The observed distribution; (B) the predicted distributions using noisy absences randomly located; (C) the predicted distributions using noisy absences following a distance gradient from the center of the environmental niche. For each noisy absence type, the top line of 3 maps refers to EM and the bottom line maps to IEM. For each line, noisy absences increase from the left to the right (left 15%, centre 45%, right 75%). The situations with 30% and 60% of noisy absences are not shown for clarity. The 100 models based on the 100 different learning data sets were used and we evaluated the percentage of mispredicting models in each pixel. The darker the pixels, the higher the percentage of prediction errors.

All the modelling iterations followed an EM process. The six statistical methods were used to build models using this learning data set (Step 2; Fig. 2). For each site, the six resulting suitabilities levels (one per modelling method) were then averaged [25], [26], [27], giving rise to a per-site suitability level [26] (Step 3; Fig. 2). We refrained from weighting the six model outputs using an accuracy measurement like the AUC, because the data set contained noisy absences. Indeed, weighting the outputs of the modelling methods could favour the models that overfit the data and hence reduce the correction rate of noisy absences. Lastly, the suitability level vector was converted into a presence-absence response, using a cut-off threshold maximizing the Kappa index (Step 4; Fig. 2). This approach was preferred to the ROC curve approach (maximising the sum of sensitivity and specificity) that gives less accurate prevalence predictions [36], [37]. These four steps account for one IEM iteration. At the first iteration, the learning data was the raw data (Step 1; Fig. 2). After the first iteration, the predicted data matrix obtained at the end of the current EM iteration was used to update the raw data set before the next iteration. To do this, observed and predicted data matrices were compared and an absence was considered as noisy when the model predicted presence while the species was absent from the observed data (i.e. the observed data was modified so that absences became presences if the model prediction was a false presence). In that case, we updated the raw data by replacing absence (0) by presence (1). The resulting data matrix was then used as the learning data set for the following iteration (Step 1; Fig. 2). The entire procedure was then repeated 100 times (Fig. 2). The modelling procedure was implemented in R [38].

### Models’ Variability

To evaluate the prediction variability inherent to the statistical methods (i.e., GBM and BT), we ran the EM 100 times for each species and each complete data set. We observed that in 95% of the cases less than 5% of the 334 test sites had variable predictions (and 11% of the sites had variable predictions). The number of different predictions was less than 27 in 95% of the cases. We thus considered that our IEM model had stabilized when less than 5% of the sites provided variable predictions in 27 successive iterations.

The evolution of the variability among the six SDM predictions through the iterative process was evaluated at each iteration. Following Thuiller [39], we carried out a standardized Principal Component Analysis (PCA) on the data matrix made up of the 6 suitability level vectors at the 334 test sites, and we evaluated the consensus among the predictions by calculating the percentage of variance accounted for by the first axis of the PCA.

**Table 1:** Null-model simulations.

		Random location of noisy absences			Gradient of noisy absences from the center of the niche		
	Percentage of noisy absences	0.01≤p<0.05	0.01≤p<0.01	p<0.001	0.01≤p<0.05	0.01≤p<0.01	p<0.001
Prevalence 15%	15%	7	8	9	5	5	15
	30%	3	0	4	1	1	8
	45%	2	0	3	1	1	4
	60%	1	2	4	2	3	3
	75%	3	1	7	2	2	11
Prevalence 30%	15%	2	5	7	1	4	5
	30%	0	1	3	2	0	1
	45%	0	0	2	0	0	1
	60%	0	0	0	0	0	0
	75%	0	1	1	0	0	1
Prevalence 60%	15%	2	1	9	3	1	11
	30%	0	0	1	0	0	0
	45%	0	0	0	0	0	0
	60%	0	0	0	0	0	0
	75%	0	0	0	0	0	0

Number of IEM models with accuracy not better than expected by chance among the 100 built on the 100 learning data sets. We counted the number of sites turned from absences to presences by the IEM procedure. The same number of sites predicted as absences by EM were randomly selected and replaced by presences. The accuracy of the resulting model was evaluated using the percentage of well-predicted sites, the TSS and Kappa indices. The accuracy was considered as lower if at least one of the three indices of the IEM was lower than that evaluated on the random predictions. The random sampling was repeated 10000 times.

The variability of the EM and IEM binary predictions inherent to the sampling of learning sites was evaluated in the same way for each virtual species, each percentage of noisy absences and each absence selection. As the 100 tests sets share a very low number of cells, we randomly selected 1000 cells among the 912730 cells covering the entire surface of France. These cells were used as a common test set for the 100 models built on the 100 learning data sets. For each of the 100 models, we predicted the presence-absence of the species over these 1000 cells. Then, we carried out a PCA on the data matrix made up of the 100 presence-absence vectors. The consensus among the predictions was evaluated by calculating the percentage of variance accounted for by the first axis of the PCA.

### Comparing IEM and EM

For each of the species, we first evaluated the AUC [40] of the mean model on the 334 test sites before using the Kappa cut-off threshold. We then evaluated the predictive accuracy of both EM and IEM presence-absence predictions on the test sites by measuring three complementary and commonly used indicators: (i) the percentage of correctly predicted sites that provides a direct measure of both true absences and true presences; (ii) the Kappa index; its dependence on prevalence merely reflects its role as a chance-corrected measure [41]; and (iii) the True Skill Statistic (TSS) which is more independent of observed species prevalence than Kappa [42]. As a complement, we assessed the ability of EM and IEM to predict the prevalence of the species by measuring the difference between the observed and the predicted prevalences. Pairwise comparisons between EM and IEM were done using Wilcoxon’s tests.

Finally, we used a null-model simulation to explore the possibility that the increase in model accuracy between EM and IEM could only be due to an increase in the predicted prevalence through the iterative process. We hence compared IEM predictive accuracy to the accuracy of the output of EM predictions modified by randomly turning some sites from absences to presences. The number of sites where absences were replaced by presences was identical to that turned from absences to presences by the IEM procedure, and we computed the percentage of mispredicted sites, Kappa and TSS. For each species, we reiterated this procedure 10 000 times for each learning data set and we compared the observed values of indices produced by the IEM with the distribution of the 10 000 values simulated by the null-model.

We also plotted a map of omission and commission errors. For each species, we predicted the presence-absence of the species over the 912730 cells covering the French territory. We then counted, over the 100 models built using the 100 different learning data sets, the percentage of mispredicting models in each cell. This was done for both EM and IEM.

## Results

### IEM Modelling

For the three species, the iterative process tended to converge rapidly, as most of the predictions stabilized after 2 to 70 iterations (mean: 15 iterations). Only 3.5% of the models did not stabilize after 70 iterations (see Fig. S1 a). The models that did not stabilize were characterized by high levels of noisy absences. Moreover, the stabilization time (i.e. number of iterations) increased with the percentage of noisy absences (Fig. S1 b).

After a few iterations, the 6 different methods provided consensual predictions for the 334 test sites (Fig. S2). At the first iteration (i.e., EM), the mean percentages of variance accounted for by the first axis of the PCA were 67.5%, 72.4% and 79.5% for the three species, respectively. Using IEM, consensus increased after 25 iterations up to 73.9%, 79%, and 86.1% respectively and then reached a relatively stable plateau up to the end of the iterative procedure (Fig. S2).

IEM also increased the consensus of predictions built on different learning data sets (Fig. S3). At the first iteration (i.e., the EM), the mean percentages of variance accounted for by the first axis of the PCA were 81.5%, 72.2%, 59.6%, 45.9% and 32.3% respectively for the five noisy absence levels. Using IEM, consensus increased up to 82%, 79.5%, 74.5%, 66.7% and 49.6% respectively. This increase was higher for frequent species especially when noisy absences were randomly selected.

### Predictive Performance

The AUC almost always significantly decreased during the iterative process but this decrease remained low except for high levels of noisy absences (Fig. 3). All AUC values were higher than 0.77 (higher than 0.88 for 95% of the models) for IEM whereas they were higher than 0.79 (higher than 0.92 for 95% of the models) for EM. Evaluating the predictive accuracy of both EM and IEM presence-absence output on the 334 test sites showed that compared with EM, IEM significantly reduced false absences (Wilcoxon test, p<0.001, Fig. 4). Due to the IEM principle (i.e., replacing noisy absences by presences in the learning data set), the model most easily predicted presences in environments that were in fact true absences, and hence false presences increased significantly in the test set predictions (Wilcoxon test, p<0.001, Fig. 4). Lowering false absences and increasing false presences led to a variation of the predictive accuracy evaluated on the test set that almost depended on the percentage of noisy absences (Fig. 4). Using IEM, the three species experienced a significant increase in predictive accuracy for noisy absences levels greater than 30% (Wilcoxon test, p<0.001). The results were more mixed for lower levels of noisy absences (15 an 30%) as both positive, negative or no change were detected between EM and IEM according to the quality index. Although some were significant, these changes remained of slight intensity (Fig. 4).

For noisy absence levels greater than 30%, iterations increased the percentage of well-predicted sites, Kappa and TSS in 93%, 97% and 84% of the cases, respectively (Fig. 4). Moreover, the Kappa index calculated for IEM gave a good score (>0.6) for 2253 out of the 3000 cases and a moderate score (between 0.4 and 0.6) for 593 cases. Our predictions were thus reliable (i.e. Kappa >0.4) in 94.9% of the cases. The performance of EM was clearly lower, with only 1376 cases reaching a Kappa score above 0.6, and 71% of the cases for which the predictions were reliable. The TSS index confirmed this trend, as TSS calculated for IEM reached a score greater than 0.6 for 73.7% of the cases and between 0.4 and 0.6 for 18.6% of the cases. TSS was lower for EM with a score greater than 0.6 for 38.8% of the cases and a score between 0.4 and 0.6 for 27% of the cases. Moreover, the IEM provided less biased estimates of species prevalence in 80.7% of the cases (96.5% of the cases with noisy absences levels greater than 30%) (Fig. 4). Note that for high levels of noisy absences, the benefit of IEM compared to EM was lower if the noisy absences were preferentially located at the edge of the niche. Moreover, species prevalence only affected the pattern at the highest noisy absence level due to the limited increase in model quality through iterations for the rarest species.

The geographical pattern of omission errors depended on the selection of noisy absences. When the noisy absences were randomly chosen, the EM mispredicted presence cells spread over the whole distribution and were slightly more abundant at the edge of the distribution. For abundant, non-random noisy absences, the EM only predicted the ‘core’ region of each species distribution. At the end of the iterative process, the remaining often omitted cells were in both cases more abundant at the edge of the distribution, but this pattern was less marked for randomly chosen noisy absences (Fig. 5, S4, S5).

The location of commission errors was less affected by the selection of noisy absences. Mispredicted absence sites were mostly located at the edge of the distribution and the IEM increased the mispredicted areas especially in areas where the environmental variables varied only slightly (Fig. 5, S4, S5).

The increase in the predictive performance between IEM and EM was not due to the rise of the predicted prevalence, as for 2963 out of the 3000 cases (1789 of the 1800 cases with levels of noisy absences greater than 30%), IEM predictions were significantly more reliable than those produced by the null-model simulations (p<0.05), considering TSS, Kappa and the percentage of well-predicted sites (Table 1).

## Discussion

In ecological sciences, the presence of an organism is factual while absence is inferred i.e., the species was not seen or identified or captured [5]. Absence is hence the main cause of uncertainty in species occurrence data matrices and thus can have detrimental consequences on the relevance of correlative SDMs [5], [6]. Alternatives are limited as the only models currently used to take species detectability into account require repeated survey data or species abundance data [9], while most data matrices are composed of presence-absence data without multiple observations. IEM provides a way to reduce this problem as it only requires presence-absence data matrices and can reduce the bias inherent in species detectability by dealing with noisy absences. Although IEM did not provide better results than the EM with low levels of noisy absences, it was significantly more efficient than EM as soon as the data set contained more than 30% of noisy absences. In such cases, it enhanced the prediction ability of correlative SDMs by increasing both the quality of the statistical models and the consensus between statistical methods. This is an important point as the variability between statistical models is recognized as the major source of uncertainty in the prediction of species spatial distributions by correlative SDMs [30]. This tendency is triggered for low detectable species [43], [44], such as species of low occurrence like large predators in forested areas [e.g. 45]. In the same way, threatened species are characterized by a high occurrence of non-contingent absences as those species have often been extirpated from a large part of their natural area. The IEM approach might therefore be of interest in the prediction of the potential distribution of threatened or difficult-to-detect species, which is not readily feasible using classical correlative SDMs [46].

Another possible application of the IEM is the prediction of the potential distribution of non-native species, which has been considered as difficult to achieve using correlative SDMs [e.g., 47,48]. Indeed, it is now recognised that most non-native species are in a non-equilibrium state, particularly due to spatial variability in propagule pressure and human impact on ecosystems across the world [49], [50]. Up until now the two ways proposed to predict the spatial invasion range of invasive species involved (1) the use of presence-only models, which have a low predictive efficiency [51], [52], or (2) the calibration of models on the niche conditions found in both the native and the exotic range of the species [17], [53], [54], with the aim of accounting for potential niche shifts between native and invasion ranges [54]–[57]. This however strongly limits the predictive efficiency of the models, as a substantial part of the absences in the exotic range are contingent, leading to an underprediction of the potential range. The IEM might hence constitute an alternative for predicting the invasion potential of current and future invaders as it has been shown to reduce omission errors that are known to be costly in the prediction of invasive species distribution, as it is more difficult to eradicate a pest than to identify a species that may become a problem [58].

As for more classical correlative SDMs, the spatial extent of presence data remains determinant in the quality of species predictions. Although IEM has been shown to be of interest in the reduction of omission errors, it should however be noted that this method remains unable to guess missing ecological information. This was observed in two ways on our virtual species for high levels of false absences. First, IEM showed less improvement in the accuracy of models built on data sets with noisy absences located at the edge of the niche. As the model did not have information on suitable environmental conditions at the edge of the environmental niche, it tended to underpredict the distribution range. Second, rare species experienced a lower accuracy increase through iteration for the highest level of noisy absence. Here, the number of observed occurrences probably fell under a critical threshold that did not permit the models to gather sufficient information to build a detailed image of the niche.

Particular attention should also be given to the selection of the environmental variables, which always remains a crucial point in the model building process [13], [53]. This is particularly true for IEM as an inaccurate variable may drive predictions in the wrong direction through iterations. For the same reason, we also warn against the use of the iterative approach when using a unique statistical method as iterations may increase bias inherent to the statistical method used, whereas the use of ensemble methods buffers potential bias due to any specific statistical method [25].

Although accidental presences (i.e. the species is recorded in environmental conditions where it is unable to settle) are usually rare in ecological data, they can occur as species misidentification in data bases or as recorded occurrences of non-established species. As IEM is designed to fill noisy absences, it may also be affected by these accidental presences. IEM might then inflate the predicted distribution by considering as noisy absences those falling in the gap between real and accidental presences. Accidental presences might therefore promote IEM niche overprediction or drive the model in the wrong direction, especially in the case of high levels of noisy absences that give more importance to the accidental presences. The effect of accidental presences on IEM hence deserves to be quantified.

In the same way, model transferability should be evaluated. We showed here that compared to EM, IEM increased the consensus between predictions based on different learning data sets. This suggests that IEM tends to reduce both the sensitivity of models to differences in the ranges of environmental predictors and the overfitting of the learning data. As these two parameters are known to reduce model transferability [59], IEM might be more transferable than EM. But EM and IEM transferability remains to be compared on real species as numerous ecological parameters are known to affect model transferability [59].

Finally, many parameters are known to affect the quality of correlative SDMs, such as the size and extent of the observed distribution, environmental parameter sampling [60], the prevalence of the species [61], cut-off selection [62], or the selection of absences used in the learning data set. The sensitivity of the IEM to these parameters remains to be evaluated before intensively using IEM. We therefore encourage complementary studies to draw up precise guidelines for the use of this method.

## Supporting Information

Figure S1 **Stabilization of the iterative process.** a) Number of sites with variable predictions during the 27 following iterations. The grey line corresponds to maximum value over the 3000 models, vertical bars correspond to the variability across 95% of the models; dots correspond to the mean values across the 3000 models. The two dashed lines correspond to the variability inherent to the statistical methods (for all the simulations and for the 95% less variable ones). b) Stabilization time (in number of iterations) of the iterative process across noisy absence levels.
(TIFF)

Figure S2 **Consensus (percentage of variance explained by the first axis of the PCA) among the six models during the iterative process for the 334 test sites.** Species prevalence (a) 15%; (b) 30%; (c) 60%. Grey lines correspond to maximum and minimum values, vertical bars correspond to the variability across 95% of the test sites; dots correspond to the mean variance.
(TIFF)

Figure S3 **Consensus (percentage of variance explained by the first axis of the PCA) among the 100 learning data sets after the first iteration (EM) and at the end of the process (IEM) for 1000 randomly selected cells over France.** Symbols represent virtual species prevalence. Circles: 15%; squares: 30%; diamonds: 60%. Colour represents noisy absence samplings. Grey: random; white: almost at the edge of the niche. Border colour represents the models. Green: EM; black: IEM.
(TIFF)

Figure S4 **The (a) observed and (b) predicted distributions of the rare species (prevalence = 15%) using noisy absences randomly located or (c) located following a distance gradient from the center of the environmental niche.** For each noisy absence type, the top line of 3 maps refers to EM and the bottom line maps to IEM. For each line, noisy absences increase from left to right (left 15%, centre 45%, right 75%). The situations with 30% and 60% of noisy absences are not shown for clarity. The 100 models based on the 100 different learning data sets were used and we evaluated the percentage of mispredicting models in each pixel. The darker the pixels, the higher the percentage of prediction errors.
(TIFF)

Figure S5 **The (a) observed and (b) predicted distributions of the intermediate species (prevalence = 30%) using noisy absences randomly located or (c) located following a distance gradient from the center of the environmental niche.** For each noisy absence type, the top line of 3 maps refers to EM and the bottom line maps to IEM. For each line, noisy absences increase from the left to the right (left 15%, centre 45%, right 75%). The situations with 30% and 60% of noisy absences are not shown for clarity. The 100 models based on the 100 different learning data sets were used and we evaluated the percentage of mispredicting models in each pixel. The darker the pixels, the higher the percentage of prediction errors.
(TIFF)
