# Supplementary figures and images for: Dealing with Noisy Absences to Optimize Species Distribution Models: An Iterative Ensemble Modelling Approach
Source: PLoS One. 2012 Nov 15;7(11):e49508. doi: 10.1371/journal.pone.0049508 (PMC3499418; doi:10.1371/journal.pone.0049508)

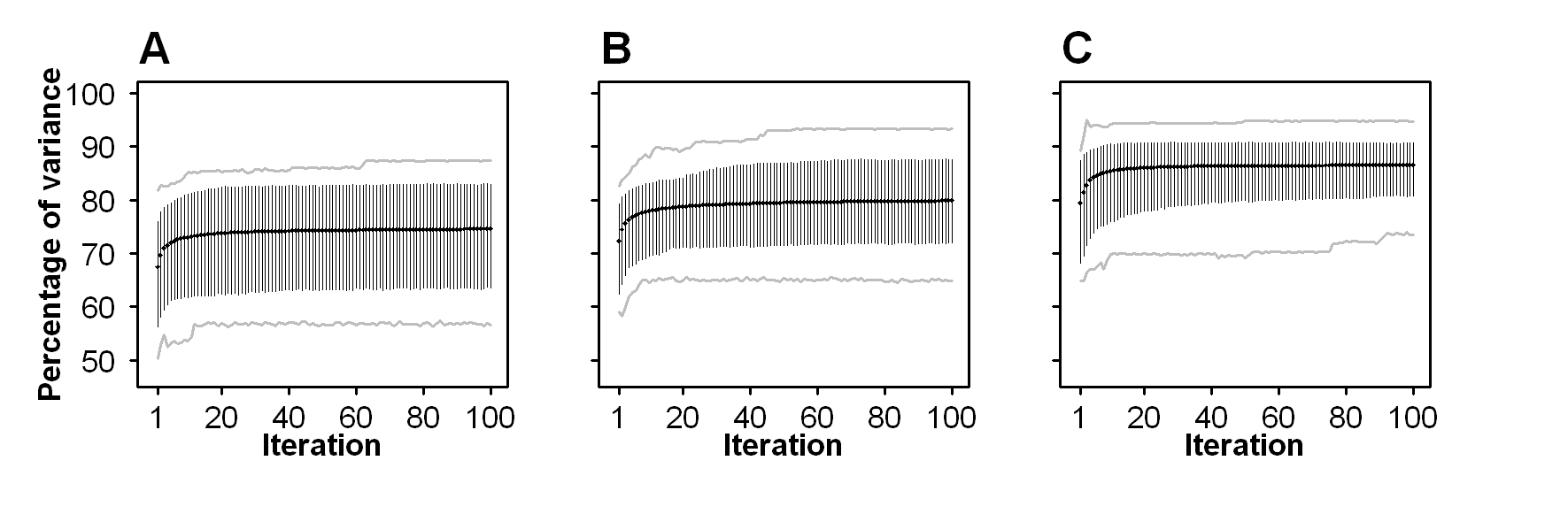

Supplement: Figure S2 — Consensus (percentage of variance explained by the first axis of the PCA) among the six models during the iterative process for the 334 test sites. Species prevalence (a) 15%; (b) 30%; (c) 60%. Grey lines correspond to maximum and minimum values, vertical bars correspond to the variability across 95% of the test sites; dots correspond to the mean variance. (TIFF) [file pone.0049508.s002.tiff]

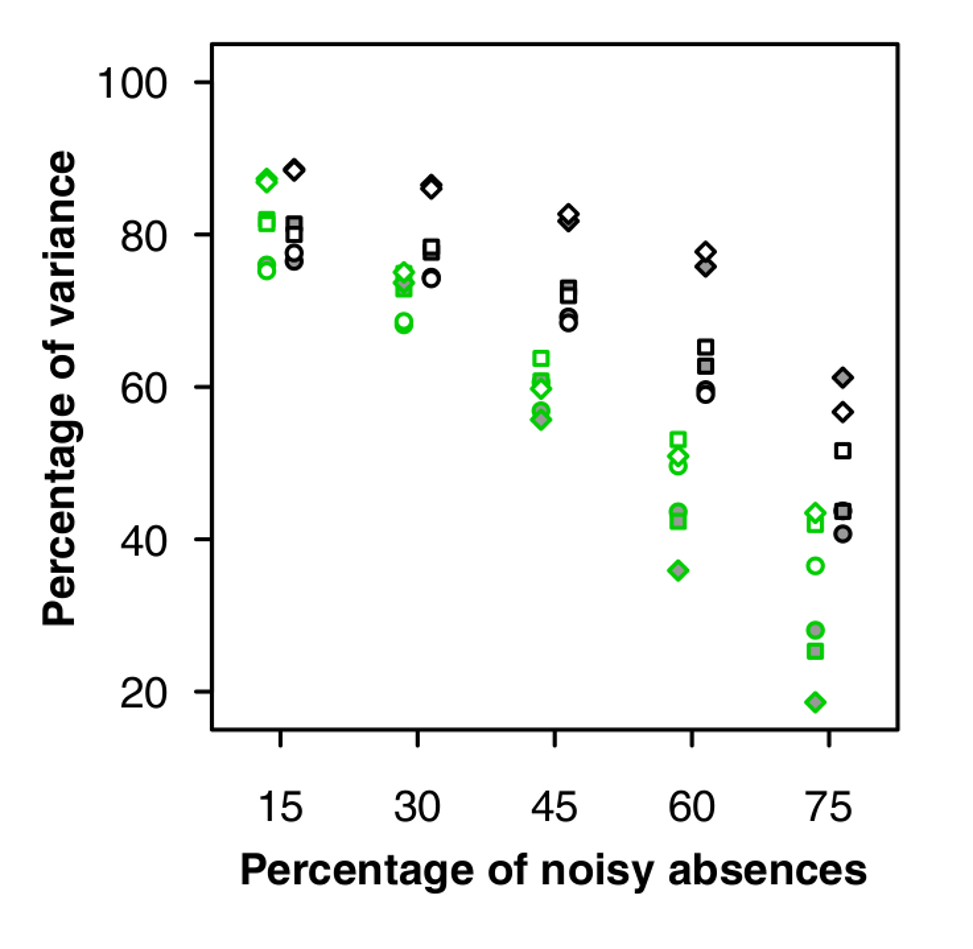

Supplement: Figure S3 — Consensus (percentage of variance explained by the first axis of the PCA) among the 100 learning data sets after the first iteration (EM) and at the end of the process (IEM) for 1000 randomly selected cells over France. Symbols represent virtual species prevalence. Circles: 15%; squares: 30%; diamonds: 60%. Colour represents noisy absence samplings. Grey: random; white: almost at the edge of the niche. Border colour represents the models. Green: EM; black: IEM. (TIFF) [file pone.0049508.s003.tiff]

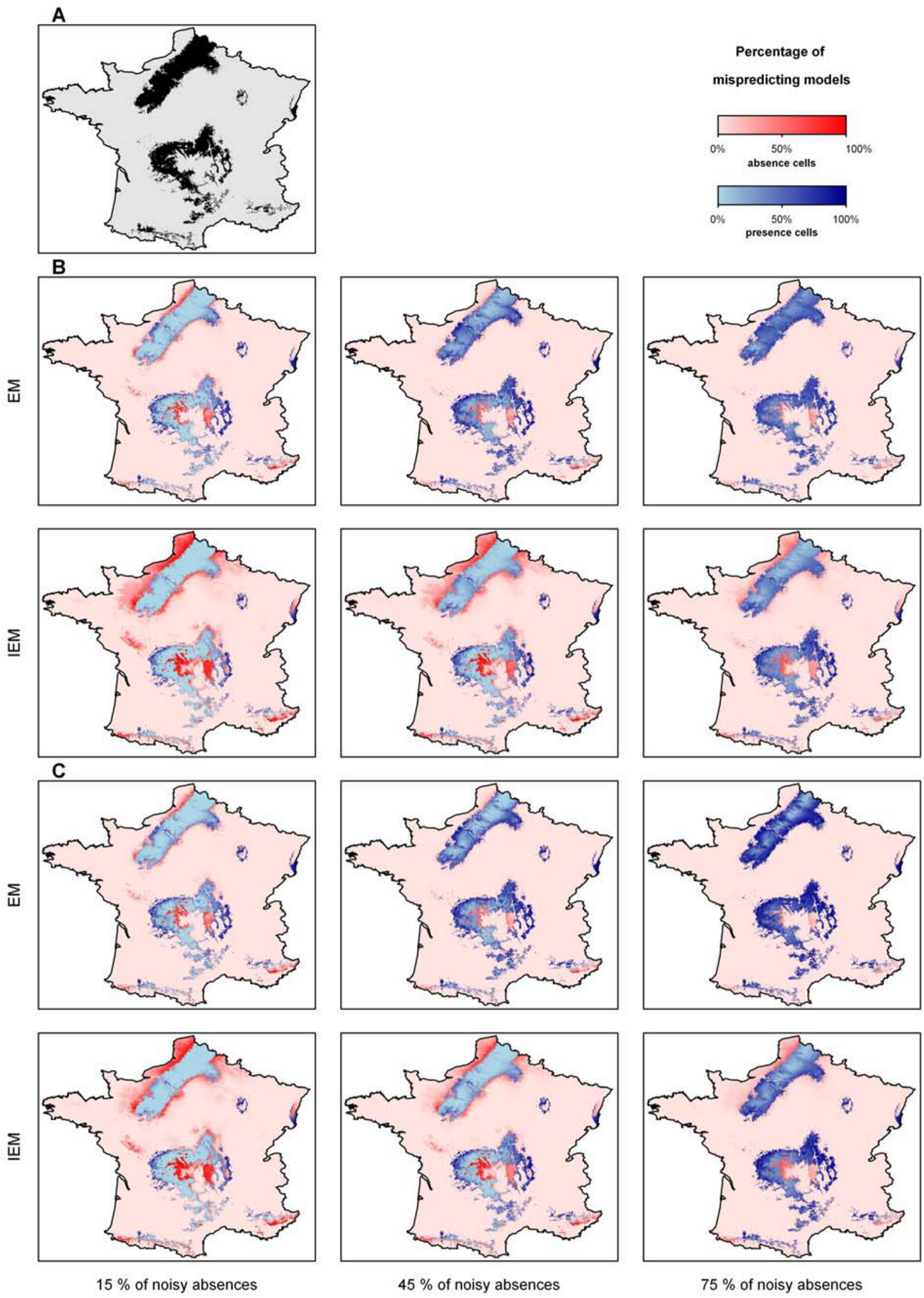

Supplement: Figure S4 — The (a) observed and (b) predicted distributions of the rare species (prevalence = 15%) using noisy absences randomly located or (c) located following a distance gradient from the center of the environmental niche. For each noisy absence type, the top line of 3 maps refers to EM and the bottom line maps to IEM. For each line, noisy absences increase from left to right (left 15%, centre 45%, right 75%). The situations with 30% and 60% of noisy absences are not shown for clarity. The 100 models based on the 100 different learning data sets were used and we evaluated the percentage of mispredicting models in each pixel. The darker the pixels, the higher the percentage of prediction errors. (TIFF) [file pone.0049508.s004.tiff]

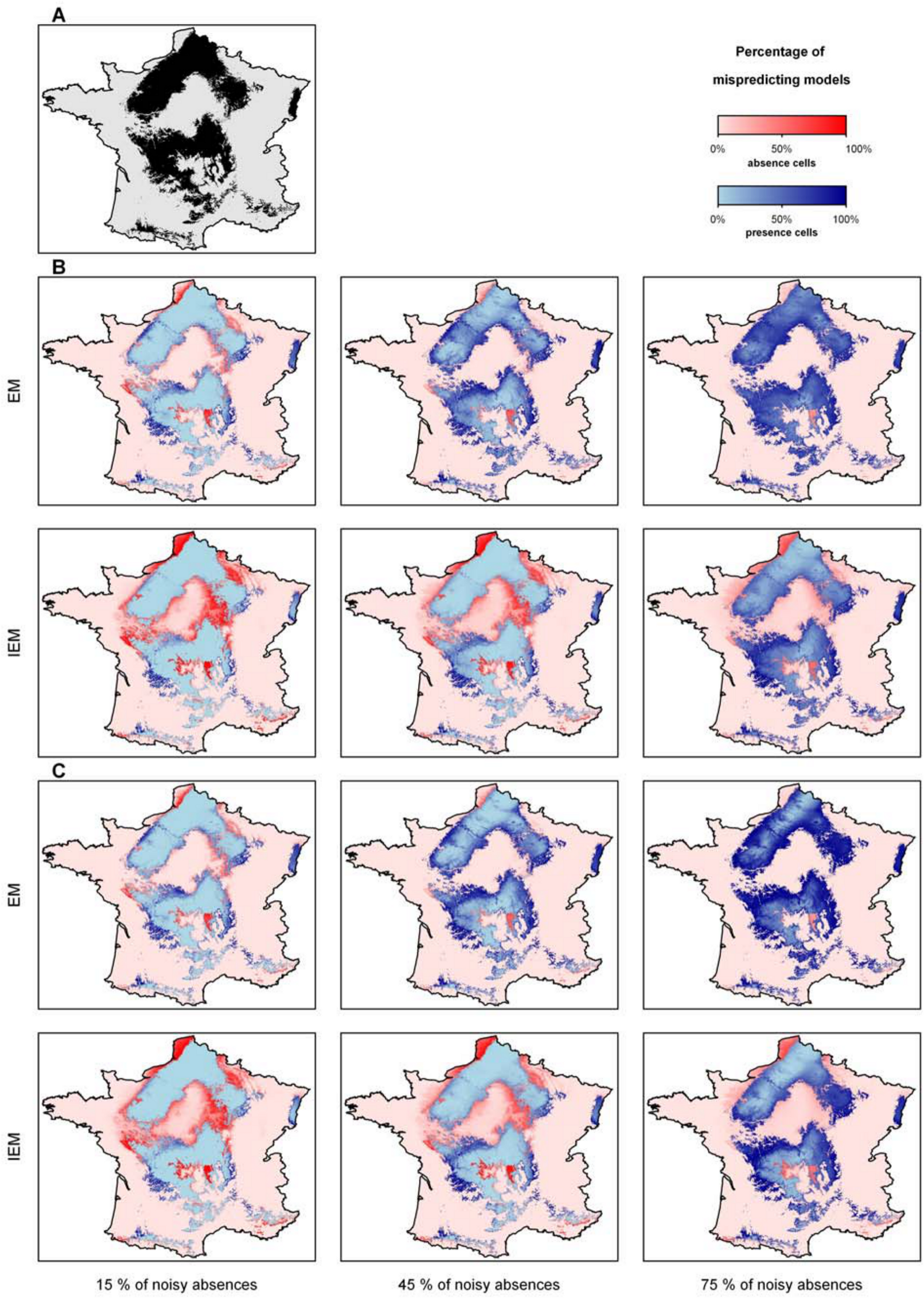

Supplement: Figure S5 — The (a) observed and (b) predicted distributions of the intermediate species (prevalence = 30%) using noisy absences randomly located or (c) located following a distance gradient from the center of the environmental niche. For each noisy absence type, the top line of 3 maps refers to EM and the bottom line maps to IEM. For each line, noisy absences increase from the left to the right (left 15%, centre 45%, right 75%). The situations with 30% and 60% of noisy absences are not shown for clarity. The 100 models based on the 100 different learning data sets were used and we evaluated the percentage of mispredicting models in each pixel. The darker the pixels, the higher the percentage of prediction errors. (TIFF) [file pone.0049508.s005.tiff]
